# Circular RNA KIF4A promotes cell migration, invasion and inhibits apoptosis through miR-152/ZEB1 axis in breast cancer

**DOI:** 10.1186/s13000-020-00963-7

**Published:** 2020-05-14

**Authors:** Yongping Jin, Liu Yang, Xia Li, Fangli Liu

**Affiliations:** 1 Institute of Nursing and Health, College of Nursing and Health of Henan University, Kaifeng, China; 2 Department of Teaching Management, School of Medicine of Kaifeng City, Kaifeng, China

**Keywords:** Breast cancer, circKIF4A, miR-152, ZEB1

## Abstract

**Background:**

Circular RNAs (circRNAs) have been demonstrated to exert crucial mediators in tumor initiation and development. Nevertheless, the roles of circKIF4A in breast cancer (BC) are still not very clear.

**Methods:**

Quantitative real-time polymerase chain reaction (qRT-PCR) was conducted to determine the expression of circKIF4A, miR-152, zinc finger E-box binding homeobox 1 (ZEB1) mRNA and caspase-3. Western blot assay was utilized to examine the protein level of ZEB1. Transwell assay and flow-cytometric analysis were adopted for the evaluation of cell migration, invasion and apoptosis, respectively. The associations among circKIF4A, miR-152 and ZEB1 were predicted by online websites and verified by dual-luciferase reporter assay and RNA immunoprecipitation (RIP) assay.

**Results:**

CircKIF4A and ZEB1 were conspicuously upregulated and miR-152 was markedly reduced in BC tissues and cells. Deficiency of circKIF4A repressed migration, invasion and induced apoptosis of BC cells. Moreover, circKIF4A was confirmed to be a sponge of miR-152 and miR-152 could bind to ZEB1. MiR-152 inhibition or ZEB1 overexpression abolished the impacts of circKIF4A knockdown on cell migration, invasion and apoptosis in BC.

**Conclusion:**

Silencing of circKIF4A hampered cell metastasis and promoted apoptosis by regulating ZEB1 via sponging miR-152 in BC.

## Introduction

Breast cancer (BC) is a frequent type of cancer with high incidence and mortality worldwide, especially in high-income countries [1, 2]. In recent years, with the improvement of treatment strategy and early detection for BC, the five-year survival of women with BC has improved [3]. However, due to the high incidence and unclear pathogenesis, the survival rate of BC patients is still not high [4]. Therefore, it is necessary to further study the molecular mechanism underlying BC and explore new targets for BC treatment.

Circular RNAs (circRNAs) are special non-coding RNAs (ncRNAs) that extensively expressed in eukaryotic cells [5]. It has been reported that circRNAs take part in many physiological and pathological processes [6]. Accumulating studies have demonstrated that the dysregulation of circRNAs participate in the regulation of malignant tumours through competitive endogenous RNAs (ceRNAs) mechanism [7]. For example, circSMARCA5 could inhibit tumor growth and metastasis in hepatocellular carcinoma by sponging miR-17-3p and miR-181b-5p [8]. CircLARP4 was demonstrated to inhibit gastric cancer cell growth and invasion through regulating the miR-424-5p/LATS1 axis [9]. CircMTO1 could decelerate hepatocellular carcinoma progression via miR-9/p21 axis [10]. All these reports indicated that circRNAs play vital roles in the carcinogenesis of human cancers. More importantly, circKIF4A regulated cell growth and motility by sponging miR-375 in triple-negative breast cancer (TNBC) [11]. Nevertheless, the functions and underlying mechanisms of circKIF4A in BC are not fully clear.

It is widely accepted that circRNAs can serve as microRNAs (miRNAs) sponges to participate in many physiological and pathophysiological processes [12]. MiRNAs are a group of small endogenous ncRNAs with about 22 nucleotides which can recognize the 3′-untranslated region (3′ UTR) of target gene to alter gene expression [13, 14]. It has been documented that multiple miRNAs are associated with the development of BC [15]. A previous study disclosed that miR-152 repressed BC cell growth and metastasis [16]. However, our understandings on the roles of miR-152 and its underlying mechanism in BC are still not enough.

Zinc finger E-box binding homeobox 1 (ZEB1) belongs to the ZEB family, and it is closely related to epithelial-to-mesenchymal transition (EMT), which is important for cell metastasis [17–19]. ZEB1 was abnormally expressed in several cancers and served as a target of miRNAs, affecting the development of cancers, including BC [20–22]. Nevertheless, the roles of ZEB1 in BC still need further investigation.

Here, the expression patterns of circKIF4A, miR-152 and ZEB1 in BC were detected. Furthermore, functional and mechanism analysis determined the exact roles and mechanisms of circKIF4A on BC cell progression.

## Materials and methods

### Tissues collection

A total of 41 BC tissues and normal tissues were harvested from patients who were diagnosed with BC at College of Nursing and Health of Henan University. All tissues were immediately placed in liquid nitrogen after removing from patients and saved at − 80 °C prior to use. The research was permitted by the Ethics Committee of College of Nursing and Health of Henan University and written informed consents were signed by all patients before surgery. None of the experimental subjects had received treatment before operation. The clinical and pathological characteristics of 41 patients with BC were shown in Table 1.

**Table 1:** Relationship between expression of circKIF4A and clinicopathological features of breast cancer patients

Clinicopathological features	Relative circKIF4A level		*P* value
	High level (%)	Low level (%)	
Age (years)			*P* > 0.05
≥ 50	16 (66.7)	8 (33.3)	
< 50	7 (41.2)	10 (58.8)	
Tumor size (cm)			*P* < 0.05
≥ 2	17 (77.3)	5 (22.7)	
< 2	6 (31.6)	13 (68.4)	
TNM stage			*P* < 0.05
I/II	8 (33.3)	16 (66.7)	
III	15 (88.2)	2 (11.8)	
ER			*P* > 0.05
Positive	7 (50)	7 (50)	
Negative	16 (59.3)	11 (40.7)	
PR			*P* > 0.05
Positive	10 (55.6)	8 (44.4)	
Negative	13 (56.5)	10 (43.5)	

### Cell culture and cell transfection

Human BC cells (MCF-7 and MDA-MB-231) and human mammary gland epithelial cells (MCF-10A) were bought from the American Type Culture Collection (ATCC, Manassas, VA, USA). MCF-7 and MDA-MB-231 cells were kept in Dulbecco’s Modified Eagle Medium (DMEM; Gibco, Carlsbad, CA, USA) including 10% fetal bovine serum (FBS; Gibco) and 1% penicillin/streptomycin (Gibco). MCF-10A cells were kept in DMEM: Nutrient Mixture F-12 (DMEM/F12; Gibco) including 5% Horse Serum (Gibco), 10 μg/mL insulin (Gibco), 20 ng/mL epidermal growth factor (Gibco) and 0.5 μg/mL hydrocortisone (Gibco) and 1% penicillin/streptomycin (Gibco). These cells were maintained in an incubator containing 5% CO_2_ at 37 °C.

Small interfering RNA (siRNA) against circKIF4A (si-circKIF4A; GCCUGGAUCUAUAACGUAUTT) and its control (si-NC; AAGTCGGGTCAAGAGAAGC), miR-152 mimics (miR-152; 5′-UCAGUGCAUGACAGAACUUGG-3′) and its control (miR-NC; 5′-GGAACUUAGCCACUGUGAAUU-3′), miR-152 inhibitors (anti-miR-152; 5′-CCAAGUUCUGUCAUGCACUGA-3′) and its control (anti-miR-NC; 5′-UCGCUUGGUGCAGGUCGGGAA-3′), pcDNA3.1-circKIF4A overexpression vector (pcDNA-circKIF4A), pcDNA3.1-ZEB1 overexpression vector (pcDNA-ZEB1) and pcDNA were bought from GenePharma (Shanghai, China). Then MCF-7 and MDA-MB-231 cells were plated into 6-well plates at a density of 1.0 × 10^5^ cells/well and transfected with indicated synthetic oligonucleotides or vectors using Lipofectamine 2000 (Invitrogen, Carlsbad, CA, USA) according to the manufacturers’ instructions. After 48 h of transfection, cells were harvested for subsequent experiments.

### Quantitative real-time polymerase chain reaction (qRT-PCR)

Total RNA was isolated using TRIzol reagent (Invitrogen). RNA concentration was quantified by NanoDrop2000 spectrophotometer (Thermo Scientific, Waltham, MA, USA). Then cDNA was synthesized by M-MLV Reverse Transcriptase Kit (Promega, Madison, WI, USA) or miRNA 1st Strand cDNA Synthesis Kit (Vazyme, Nanjing, China). QRT-PCR was carried out using an iQ™ SYBR® Green Supermix (Bio-Rad Laboratories, Philadelphia, PA, USA). The expression of circKIF4A, ZEB1, Caspase-3 and miR-152 was analyzed using the 2^-ΔΔCt^ method with GAPDH or U6 as an internal control [23]. The primers used in our study were purchased from GeneCopoeia (Guangzhou, China) and primers sequences were: circKIF4A: (F: 5′-GAGGTACCCTGCCTGGATCT-3′ and R: 5′-TGGAATCTCTGTAGGGCACA-3′); ZEB1: (F: 5′-TCCTCGAGGCACCTGAAGAGG-3′ and R: 5′-CAGAGAGGTAAAGCGTTTATAGCC-3′); Caspase-3: (F: 5′-TGGAACGAACGGACCTGTG-3′ and R: 5′-CGGGTGCGGTAGAGTAAGC-3′); miR-152: (F: 5′-GTGCAGGGTCCGAGGT-3′ and R: 5′- TGACAGAACTTGGGTCGT-3′); GAPDH: (F: 5′-TGTTCGTCATGGGTGTGAAC-3′ and R: 5′-ATGGCATGGACTGTGGTCAT-3′); U6: (F: 5′-CGCTTCGGCAGCACATATAC-3′ and R: 5′-TTCACGAATTTGCGTGTCAT-3′).

### Transwell assay

BC cells were re-suspended in DMEM (Gibco) at a density of 5.0 × 10^4^ cells. For the detection of cell migration, 300 μL cell suspension was placed into the upper chamber of a transwell (8 μm pore; Corning Incorporated, Corning, NY, USA) and the bottom chamber was filled with 300 μL DMEM (Gibco) including 10% FBS (Gibco). After incubation for 24 h at 37 °C, cells that passed through the membranes were treated with 4% para-formaldehyde (PFA) and stained with 0.1% crystal violet (Solarbio, Beijing, China). Then the migrated cells were counted under a microscope (Olympus, Tokyo, Japan). For the detection of cell invasion, the steps were the same as above, except that the upper chamber was pre-coated Matrigel (Corning Life Sciences, Corning, NY, USA).

### Flow-cytometric analysis

Cell apoptosis was evaluated by an Annexin V-fluorescein isothiocyanate (FITC)/propidium iodide (PI) apoptosis detection kit (Vazyme). Briefly, collected cells were washed, re-suspended and then stained with 5 μL Annexin V-FITC and PI for 15 min at room temperature in the dark. Apoptotic cells were analyzed within 1 h by the flow cytometry (BD Biosciences, San Jose, CA, USA).

### Dual-luciferase reporter assay

The sequences of circKIF4A (or 3′ UTR of ZEB1) including the putative complementary sequences of wild-type or mutant miR-152 were cloned into pmirGLO vectors (Promega) to generate luciferase reporter plasmids circKIF4A-WT, circKIF4A-MUT, ZEB1-WT and ZEB1-MUT, respectively. MiR-152 or miR-NC and corresponding vector were co-transfected into BC cells. After 48 h, the luciferase activity was examined using the Dual-Luciferase Reporter Assay Kit (Promega).

### RNA immunoprecipitation (RIP) assay

RNA immunoprecipitation (RIP) assay was conducted using an EZMagna RIP kit (Millipore, Billerica, MA, USA). MiR-152 or miR-NC transfected BC cells were harvested and lysed in RIP buffer. Then cell lysates were interacted with magnetic beads conjugated with Ago2 or IgG antibody. After RNAs were isolated, the enrichment of circKIF4A and ZEB1 was examined via qRT-PCR.

### Western blot assay

Total protein was extracted from BC tissues and cells with RIPA buffer (Beyotime, Shanghai, China) and determined using a BCA Protein Assay Kit (Beyotime). Equal amount of proteins was separated by SDS-PAGE and transferred to PVDF membranes (Millipore). Then the membranes were blocked with 5% nonfat milk at room temperature for 1 h and incubated corresponding primary antibodies against ZEB1 (1:2000; Santa Cruz, Dallas, TX, USA) or β-actin (1:5000; Santa Cruz) overnight at 4 °C with and horseradish peroxidase-conjugated secondary antibody (1:5000; Santa Cruz) for 1 h at room temperature. At last, protein bands were visualized by an enhanced chemiluminescence chromogenic substrate (Beyotime).

### Statistical analysis

All data were exhibited as the means ± standard deviation (SD) from at least three independent experiments. The differences were analyzed by Student’s *t*-test or one-way analysis of variance (ANOVA). The association between circKIF4A level and clinicopathologic features of patients was analyzed by χ^2^ test. It was defined as statistically difference if *P* value less than 0.05.

## Results

### CircKIF4A was increased in human BC tissues and cells

**Fig. 1:**
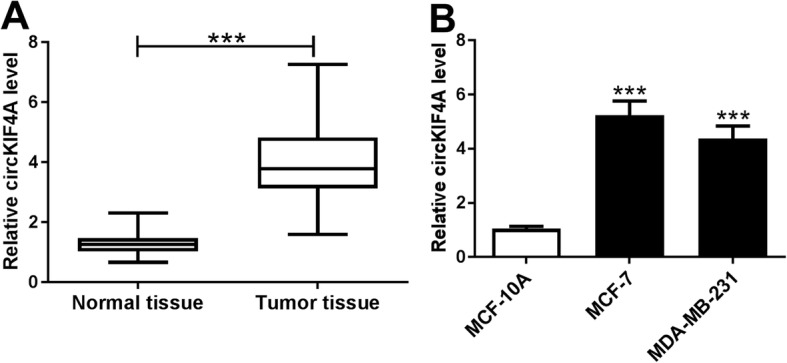
High expression of circKIF4A in BC tissues and cells. (**a**) The level of circKIF4A in BC tissues and normal tissues was evaluated using qRT-PCR. (**b**) The expression of circKIF4A in MCF-7, MDA-MB-231 and MCF-10A cells was determined using qRT-PCR. ***P < 0.001

To explore the potential role of circKIF4A in BC progression, we first determined the level of circKIF4A in 41 BC tissues and corresponding normal tissues by qRT-PCR. As we observed, circKIF4A was markedly elevated in BC tissues in reference to normal tissues (Fig. 1a). Subsequently, the level of circKIF4A in two BC cell lines and one normal breast cell line was detected. The results exhibited that circKIF4A was obviously raised in MCF-7 and MDA-MB-231 cells compared to that in MCF-10A cells (Fig. 1b). Thus, we thought that the dysregulation of circKIF4A might be involved in the development of BC.

### Silencing of circKIF4A repressed cell migration, invasion and facilitated apoptosis in BC

**Fig. 2:**
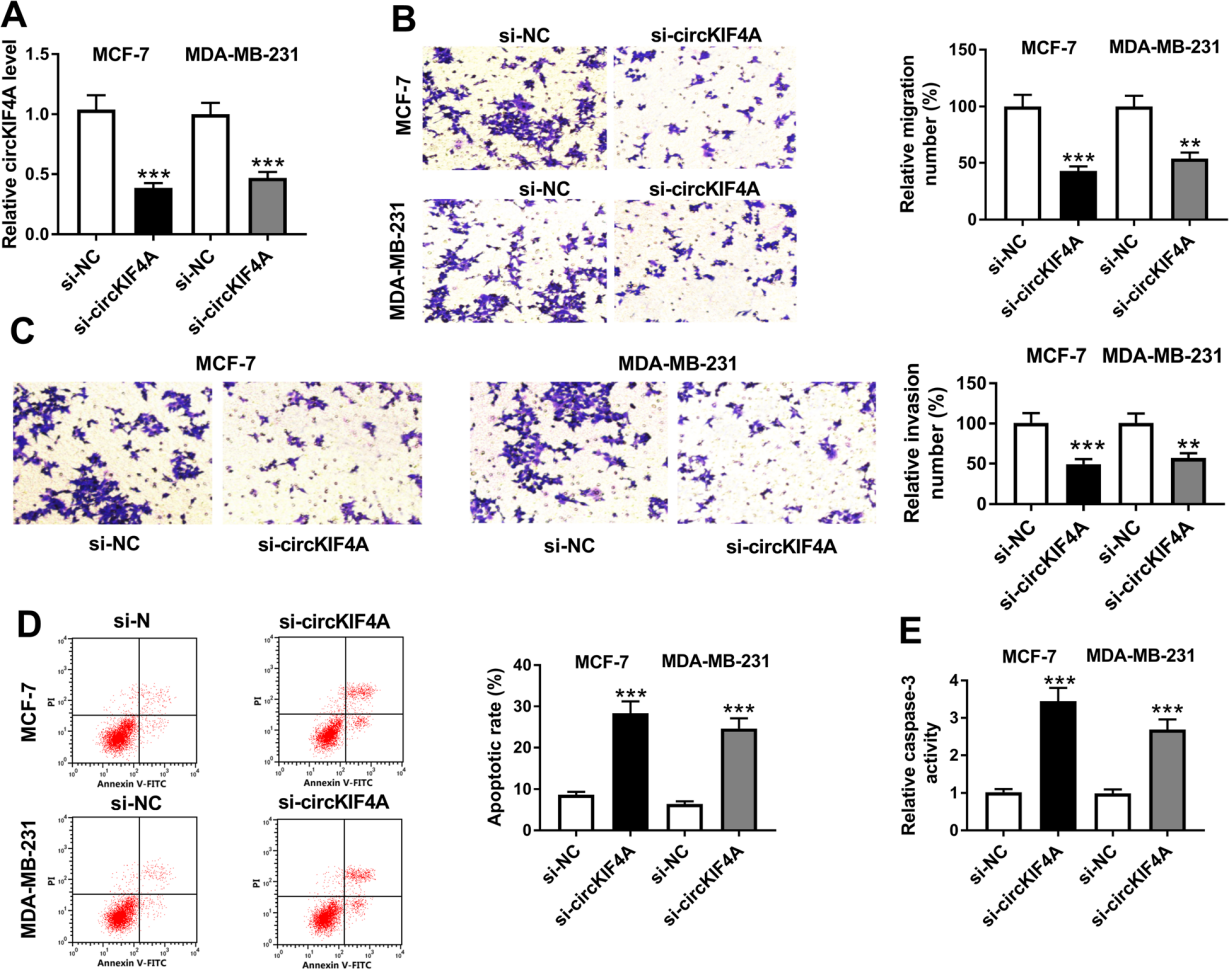
CircKIF4A knockdown hampered BC cell migration, invasion and promoted apoptosis. MCF-7 and MDA-MB-231 cells were treated with si-circKIF4A or si-NC. (**a**) CircKIF4A level was measured using qRT-PCR. (**b, c and d**) Cell migration, invasion and apoptosis were detected by transwell assay or flow cytometry analysis. (**e**) The level of caspase-3 was measured via qRT-PCR. **P < 0.01, ***P < 0.001

In order to investigate the functions of circKIF4A in the development of BC in vitro, si-circKIF4A was transfected into BC cells to knockdown the expression of circKIF4A. Knockdown efficiency was detected by qRT-PCR, and we found that circKIF4A was conspicuously decreased in si-circKIF4A transfected MCF-7 and MDA-MB-231 cells compared with si-NC group (Fig. 2a). Furthermore, transwell assay indicated that cell migration and invasion were markedly decreased in si-circKIF4A transfected group in reference to control group in MCF-7 and MDA-MB-231 cells (Fig. 2b and c). Flow-cytometric analysis showed that circKIF4A silencing led to a marked enhancement of cell apoptosis in MCF-7 and MDA-MB-231 cells compared to NC group (Fig. 2d). Then the level of caspase-3 was determined by qRT-PCR, we found that si-circKIF4A transfection caused an obvious elevation of caspase-3 in MCF-7 and MDA-MB-231 cells (Fig. 2e). These data implicated that circKIF4A knockdown suppressed BC cell progression.

### CircKIF4A modulated miR-152 expression by direct interaction in BC cells

**Fig. 3:**
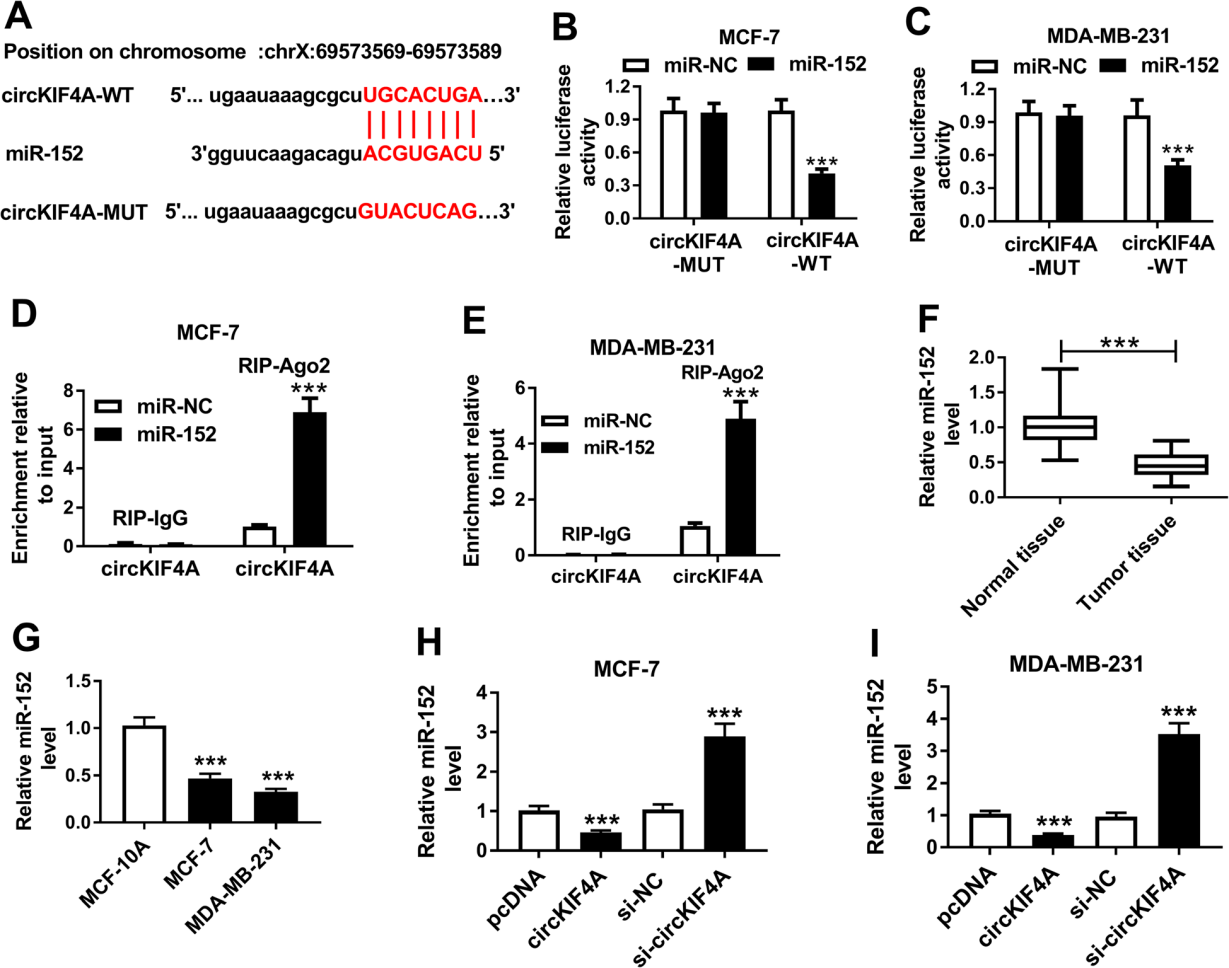
CircKIF4A interacted with miR-152 and negatively regulated its expression. (**a**) The putative binding sequences between circKIF4A and miR-152 were shown. (**b and c**) The association between circKIF4A and miR-152 was analyzed using dual-luciferase reporter assay. (**d and e**) RIP and qRT-PCR assays were performed to measure the binding efficiency of circKIF4A and miR-152 to Ago2 or IgG in MCF-7 and MDA-MB-231 cells. (**f and g**) The expression of miR-152 in BC tissues and cells was determined by qRT-PCR. (**h and i**) The level of miR-152 in BC cells transfected with pcDNA-circKIF4A or si-circKIF4A or their controls was detected by qRT-PCR. ***P < 0.001

It is widely accepted that circRNAs can modulate gene expression by sponging miRNAs [24]. By searching online website starBase v2.0 (http://starbase.sysu.edu.cn/), circKIF4A was found to contain the binding sequences of miR-152 (Fig. 3a). To confirm this prediction, dual-luciferase reporter assay was firstly conducted. The outcomes suggested that the luciferase activity was obviously reduced in circKIF4A-WT and miR-152 co-transfected cells compared to circKIF4A-WT and miR-NC co-transfected groups, whereas the luciferase activity was not changed in circKIF4A-MUT group (Fig. 3b and c). Then, RIP assay was further carried to verify the interaction between circKIF4A and miR-152. The data showed that the enrichment of circKIF4A was greatly increased in BC cells transfected with miR-152 (Fig. 3d and e), which further confirmed our prediction. As we expected, miR-152 was drastically downregulated in BC tissues and cells relative to normal tissues and cells (Fig. 3f and g). Furthermore, we determined the level of miR-152 in MCF-7 and MDA-MB-231 cells transfected with pcDNA-circKIF4A or si-circKIF4A. The results indicated that miR-152 was markedly downregulated by circKIF4A overexpression, whereas miR-152 expression was significantly upregulated after si-circKIF4A transfection in both MCF-7 and MDA-MB-231 cells (Fig. 3h and i). Collectively, circKIF4A directly interacted with miR-152 and suppressed miR-152 expression in BC cells.

### CircKIF4A regulated ZEB1 expression by targeting miR-152 in BC cells

**Fig. 4:**
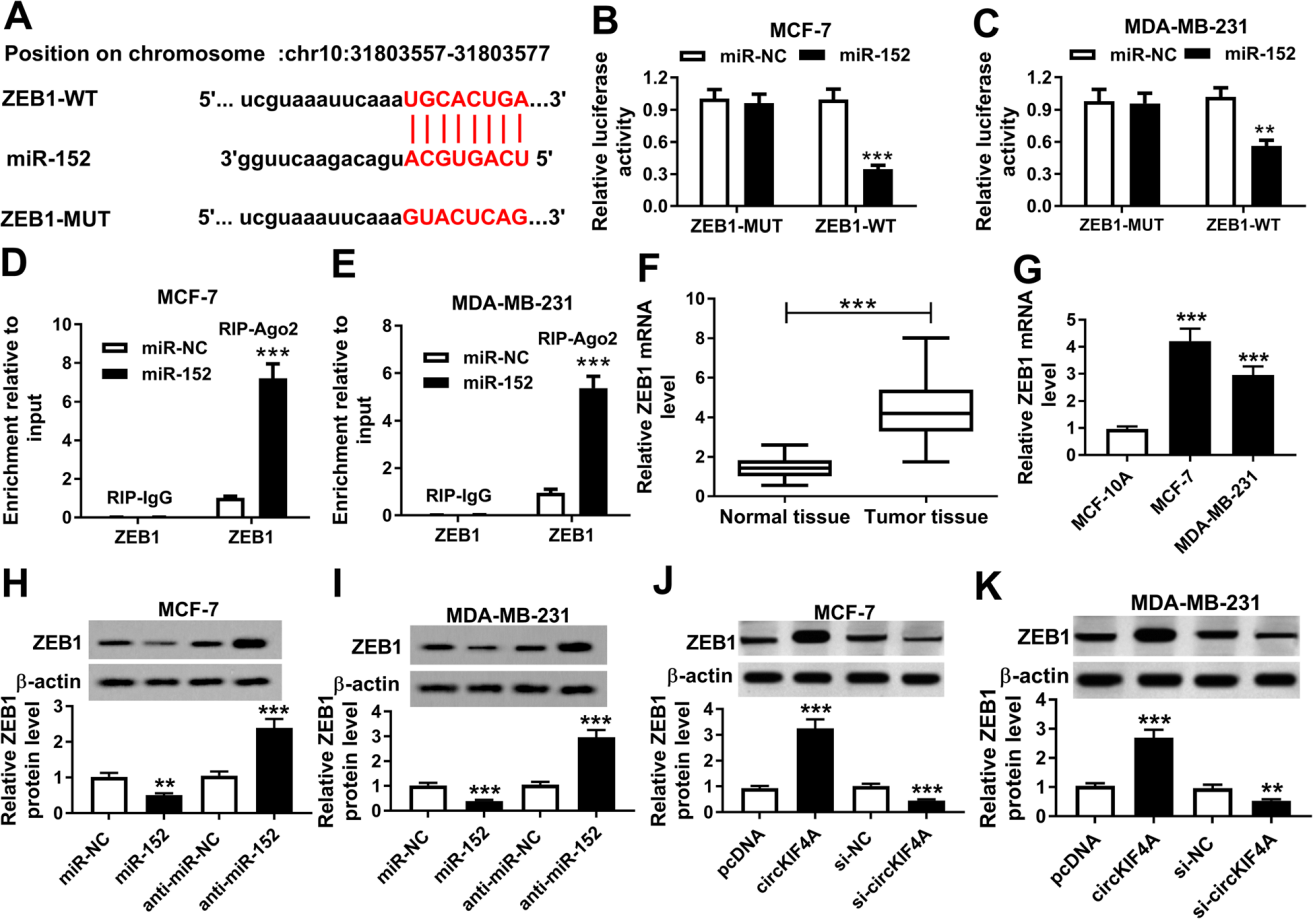
CircKIF4A regulated ZEB1 expression by targeting miR-152. (**a**) The predicted binding sites between miR-152 and 3′ UTR of ZEB1. (**b and c**) The luciferase activity was detected in MCF-7 and MDA-MB-231 cells transfected with ZEB1 3′ UTR-WT or ZEB1 3′ UTR-MUT and miR-152 or miR-NC. (**d and e**) RIP assay was conducted, and the enrichment of ZEB1 was detected by qRT-PCR. (**f and g**) The mRNA level of ZEB1 in BC tissues and cells was determined via qRT-PCR. (**h and i**) The protein level of ZEB1 in BC cells treated with miR-152, anti-miR-152 or their controls was detected by western blot assay. (**j and k**) The protein level of ZEB1 in BC cells treated with circKIF4A, si-circKIF4A or their corresponding controls was detected by western blot assay. **P < 0.01, ***P < 0.001

By searching online software DINAN tool (http://diana.imis.athena-innovation.gr/DianaTools/index.php), the potential target of miR-152 was predicted. It showed that 3′ UTR of ZEB1 might contain the complementary sequences of miR-152 (Fig. 4a). To confirm it, dual-luciferase reporter assay was performed. Our results presented that the luciferase activity was markedly inhibited in both MCF-7 and MDA-MB-231 cells co-transfected with ZEB1-WT and miR-152, while there was no significant difference in ZEB1-MUT groups (Fig. 4b and c). RIP assay presented that the enrichment of ZEB1 was distinctly increased in MCF-7 and MDA-MB-231 cells treated with miR-152 (Fig. 4d and e). Moreover, we observed that ZEB1 mRNA was elevated in BC tissues and cells in reference to that in normal tissues and cells (Fig. 4f and g). Subsequently, the protein level of ZEB1 in MCF-7 and MDA-MB-231 cells treated with miR-152, anti-miR-152, circKIF4A, si-circKIF4A or their corresponding controls was measured using western blot assay. The results implicated that miR-152 obviously suppressed ZEB1 expression and miR-152 inhibition significantly increased ZEB1 expression in both MCF-7 and MDA-MB-231 cells (Fig. 4h and i). Overexpression of circKIF4A resulted in a remarked increase of ZEB1 and knockdown of circKIF4A resulted in a significant decrease of ZEB1 in MCF-7 and MDA-MB-231 cells (Fig. 4j and k). To sum up, circKIF4A positively regulated ZEB1 expression through targeting miR-152 in BC cells.

### Inhibition of miR-152 restored the impacts of circKIF4A silencing on cell migration, invasion and apoptosis in BC cells

**Fig. 5:**
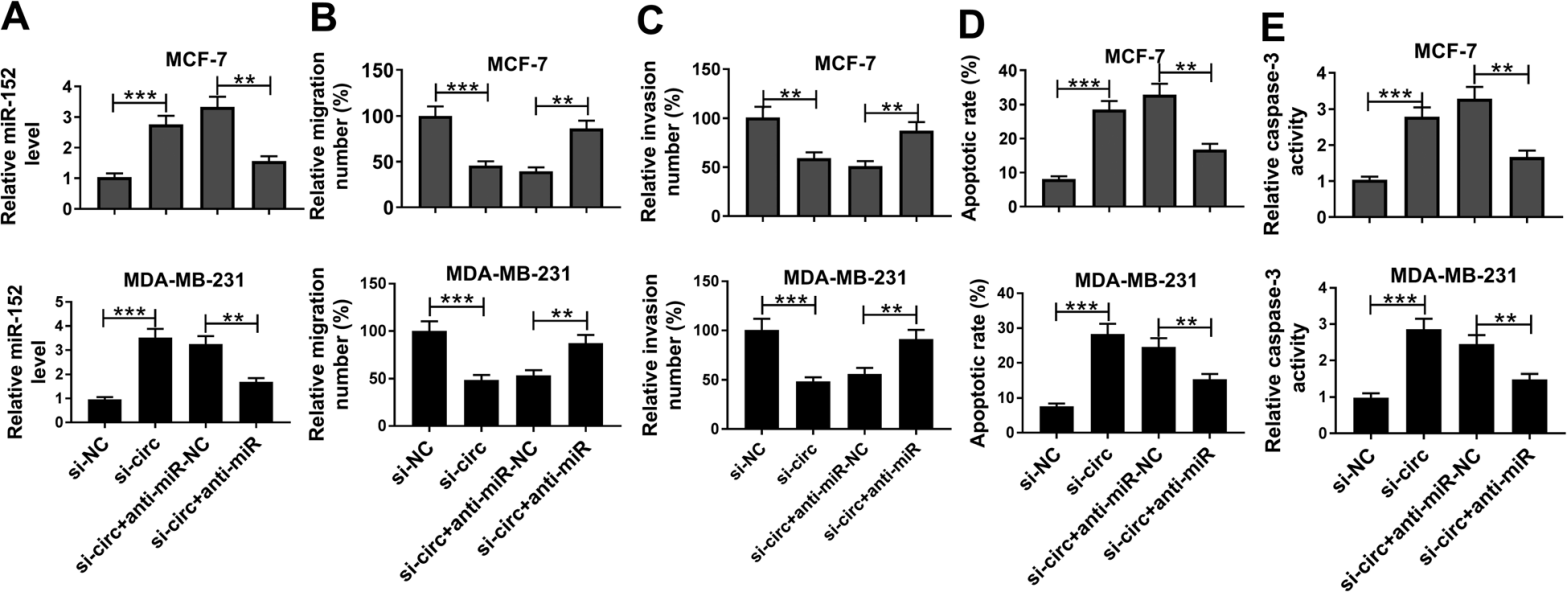
Depletion of miR-152 weakened the impacts of circKIF4A knockdown on migration, invasion and apoptosis of BC cells. MCF-7 and MDA-MB-231 cells were assigned to si-circKIF4A, si-NC, si-circKIF4A + anti-miR-152 and si-circKIF4A + anti-miR-NC groups. (**a**) The expression of miR-152 was detected by qRT-PCR. (**b and c**) Cell migration and invasion were evaluated by transwell assay. (**d**) Cell apoptosis ability was evaluated using flow cytometry assay. (**e**) Caspase-3 level was examined by qRT-PCR. **P < 0.01, ***P < 0.001

To further illustrate the relationship between circKIF4A and miR-152 in BC, MCF-7 and MDA-MB-231 cells transfected with si-NC, si-circKIF4A, si-circKIF4A + anti-miR-152 or si-circKIF4A + anti-miR-NC. As presented in Fig. 5a, the upregulation of miR-152 caused by si-circKIF4A transfection was partially restored by miR-152 inhibition in MDA-MB-231 and MCF-7 cells. Transwell assay indicated that the migration and invasion of MCF-7 and MDA-MB-231 cells were effectively suppressed by si-circKIF4A transfection, while the effects could be abolished by anti-miR-152 transfection (Fig. 5b and c). Flow-cytometric analysis indicated that circKIF4A knockdown significantly induced the apoptosis of MCF-7 and MDA-MB-231 cells, whereas depletion of miR-152 could partially abolish the effect (Fig. 5d). Moreover, the increased level of caspase-3 caused by circKIF4A knockdown was partly attenuated by miR-152 inhibition in MCF-7 and MDA-MB-231 cells (Fig. 5e). Thus, we concluded that circKIF4A silencing could decelerate BC cell progression by interacting with miR-152.

### ZEB1 overexpression weakened the influences of circKIF4A deficiency on cell migration, invasion and apoptosis in BC

**Fig. 6:**
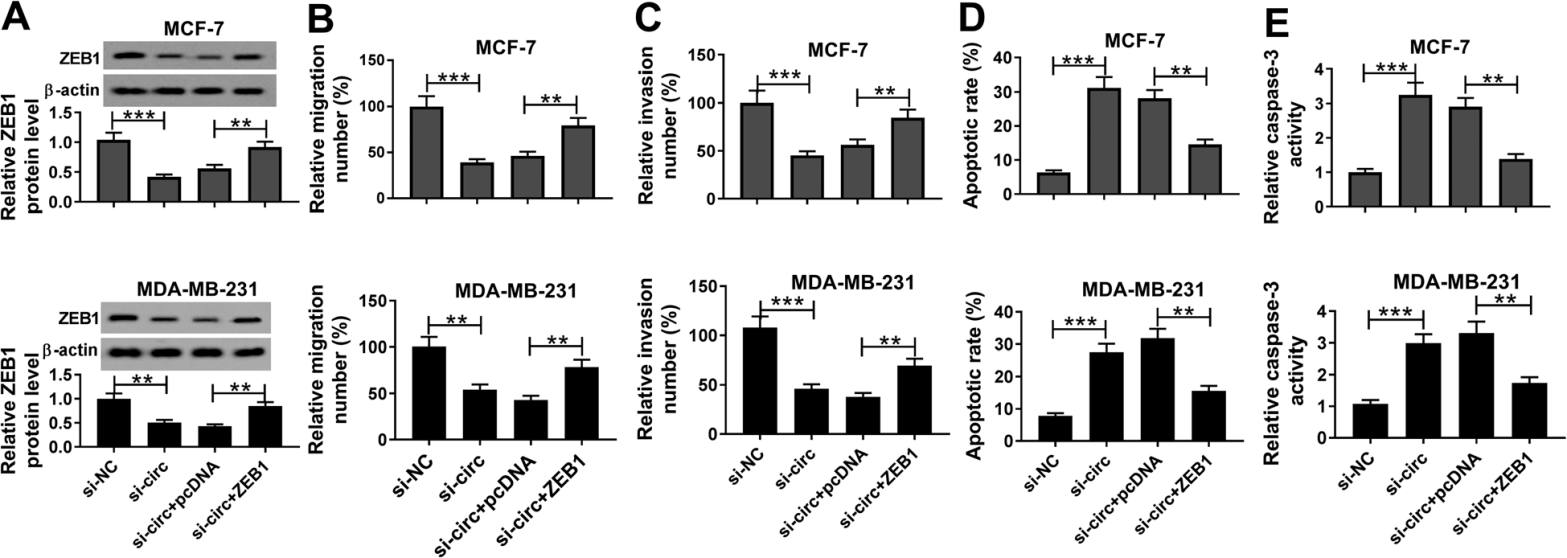
ZEB1 overexpression rescued the impacts of circKIF4A silencing on cell migration, invasion and apoptosis in BC cells. MCF-7 and MDA-MB-231 cells were treated with si-circKIF4A, si-NC, si-circKIF4A + ZEB1 or si-circKIF4A + pcDNA. (**a**) The expression of ZEB1 was measured through western blot assay. (**b and c**) Cell migration and invasion were assessed by transwell assay. (**d**) Cell apoptosis ability was detected by flow cytometry. (**e**) The level of caspase-3 was evaluated by qRT-PCR. **P < 0.01, ***P < 0.001

To further investigate the relationship between circKIF4A and ZEB1, MCF-7 and MDA-MB-231 cells were assigned to si-NC, si-circKIF4A, si-circKIF4A + pcDNA and si-circKIF4A + ZEB1. As displayed in Fig. 6a, si-circKIF4A led to a reduction of ZEB1 in MCF-7 and MDA-MB-231 cells, while the elevation of ZEB1 overturned the impact. Then we evaluated the migration, invasion and apoptosis of MCF-7 and MDA-MB-231 cells by transwell assay or flow-cytometric analysis The results displayed that the suppressive roles in cell migration and invasion and the promotional role in apoptosis in MCF-7 and MDA-MB-231 cells mediated by circKIF4A silencing were also partially rescued following the treatment of ZEB1 (Fig. 6b, c and d). Additionally, the elevation of caspase-3 caused by si-circKIF4A transfection was decreased by ZEB1 overexpression in both MCF-7 and MDA-MB-231 cells (Fig. 6e). These data illustrated that circKIF4A affected BC progression by regulating ZEB1 expression.

## Discussion

BC is the most common type of cancer in women [25]. Emerging studies indicated that circRNAs play crucial roles in several human cancers, including BC [26, 27]. Here, we mainly investigated the roles of circKIF4A and its underlying mechanisms in BC. A previous document revealed that circKIF4A was significantly elevated and circKIF4A inhibition suppressed cell growth and motility in TNBC [11]. Consistent with this finding, our study exhibited that circKIF4A was markedly elevated in BC tissues and cells. CircKIF4A deficiency markedly repressed cell metastasis and promoted apoptosis in BC. Besides, we found caspase-3 was upregulated in BC cells after circKIF4A knockdown. Caspase-3 is an executive molecule, which plays the function of apoptosis in various apoptotic pathways [28]. These results revealed that circKIF4A played important roles in the progression of BC. Therefore, we speculated that circKIF4A might act as a therapeutic target for BC.

Increasing evidence has demonstrated that circRNAs can function as miRNAs sponges to bind to miRNAs [24]. In our research, we found that circKIF4A contained the binding sites for miR-152 and miR-152 was distinctly reduced in BC tissues and cells. It has been verified that miRNAs are dysregulated in several of cancers and act as essential regulators in cancer development [29]. Indeed, miR-152 was decreased in some cancers, such as hepatocellular carcinoma [30], prostate cancer [31], colorectal cancer [32] and cervical cancer [33]. Moreover, a previous study showed that miR-152 was obviously reduced in BC, and miR-152 overexpression significantly hampered the metastasis of BC cells [16]. Our study showed that circKIF4A overexpression significantly decreased miR-152 expression, while circKIF4A knockdown significantly increased miR-152 expression. Furthermore, inhibition of miR-152 restored the influences of circKIF4A knockdown on BC cell metastasis and apoptosis.

ZEB1 is an EMT inducing transcription factor, and it is critical for tumor cell invasion and dissemination [17]. ZEB1 has been demonstrated to be abnormally expressed in several cancers and served as a target of miRNAs [20–22]. In our current study, ZEB1 was a target of miR-152 and circKIF4A upregulated the expression of ZEB1 by inhibiting miR-152 expression in BC cells. Additionally, ZEB1 overexpression abolished the impacts of circKIF4A deficiency on BC cell metastasis and apoptosis. These findings indicated that circKIF4A promoted cell migration, invasion and inhibited apoptosis by positively regulating ZEB1 expression via sponging miR-152.

However, there were still some defects in our study. For example, the tissue samples were insufficient. Moreover, we did not verify our results in vivo experiments. We will perform the experiments in our further study.

## Conclusion

Taken together, our study disclosed that circKIF4A was conspicuously elevated in BC. CircKIF4A contributed cell metastasis and hampered apoptosis by miR-152/ZEB1 axis in BC. This study manifested that circKIF4A might be a promising therapeutic target for patients with BC.

## Data Availability

The analyzed data sets generated during the present study are available from the corresponding author on reasonable request.

## References

[1] Bray F, Soerjomataram I (2015). The changing global burden of cancer: transitions in human development and implications for cancer prevention and control. Cancer.

[2] Newman LA (2015). Disparities in breast cancer and african ancestry: a global perspective. Breast J.

[3] Xue J, Chi Y, Chen Y, Huang S, Ye X, Niu J, Wang W, Pfeffer LM, Shao Z, Wu ZH (2016). MiRNA-621 sensitizes breast cancer to chemotherapy by suppressing FBXO11 and enhancing p53 activity. Oncogene..

[4] Cedolini C, Bertozzi S, Londero AP, Bernardi S, Seriau L, Concina S, Cattin F, Risaliti A (2014). Type of breast cancer diagnosis, screening, and survival. Clin Breast Cancer.

[5] Jeck WR, Sharpless NE (2014). Detecting and characterizing circular RNAs. Nat Biotechnol.

[6] Mercer TR, Mattick JS (2013). Structure and function of long noncoding RNAs in epigenetic regulation. Nat Struct Mol Biol.

[7] Kulcheski FR, Christoff AP, Margis R (2016). Circular RNAs are miRNA sponges and can be used as a new class of biomarker. J Biotechnol.

[8] Yu J, Xu QG, Wang ZG, Yang Y, Zhang L, Ma JZ, Sun SH, Yang F, Zhou WP (2018). Circular RNA cSMARCA5 inhibits growth and metastasis in hepatocellular carcinoma. J Hepatol.

[9] Zhang J, Liu H, Hou L, Wang G, Zhang R, Huang Y, Chen X, Zhu J (2017). Circular RNA_LARP4 inhibits cell proliferation and invasion of gastric cancer by sponging miR-424-5p and regulating LATS1 expression. Mol Cancer.

[10] Han D, Li J, Wang H, Su X, Hou J, Gu Y, Qian C, Lin Y, Liu X, Huang M (2017). Circular RNA circMTO1 acts as the sponge of microRNA-9 to suppress hepatocellular carcinoma progression. Hepatology..

[11] Tang H, Huang X, Wang J, Yang L, Kong Y, Gao G, Zhang L, Chen ZS, Xie X (2019). circKIF4A acts as a prognostic factor and mediator to regulate the progression of triple-negative breast cancer. Mol Cancer.

[12] Hansen TB, Jensen TI, Clausen BH, Bramsen JB, Finsen B, Damgaard CK, Kjems J (2013). Natural RNA circles function as efficient microRNA sponges. Nature..

[13] Ambros V (2001). microRNAs: tiny regulators with great potential. Cell..

[14] Hua Y, Duan S, Murmann AE, Larsen N, Kjems J, Lund AH, Peter ME (2011). miRConnect: identifying effector genes of miRNAs and miRNA families in cancer cells. PloS one.

[15] O'Day E, Lal A (2010). MicroRNAs and their target gene networks in breast cancer. Breast Cancer Res.

[16] Maimaitiming A, Wusiman A, Aimudula A, Kuerban X, Su P (2020). microRNA-152 inhibits cell proliferation, migration and invasion in breast cancer. Oncol Res.

[17] Bakiri L, Macho-Maschler S, Custic I, Niemiec J, Guio-Carrion A, Hasenfuss S, Eger A, Müller M, Beug H, Wagner E (2015). Fra-1/AP-1 induces EMT in mammary epithelial cells by modulating Zeb1/2 and TGFβ expression. Cell Death Differ.

[18] Schmalhofer O, Brabletz S, Brabletz T (2009). E-cadherin, β-catenin, and ZEB1 in malignant progression of cancer. Cancer Metastasis Rev.

[19] Sekido R, Takagi T, Okanami M, Moribe H, Yamamura M, Higashi Y, Kondoh H (1996). Organization of the gene encoding transcriptional repressor δEF1 and cross-species conservation of its domains. Gene..

[20] Gong J, Wang Y, Jiang B, Xu B, Hu C (2019). Impact of high-mobility-group A2 overexpression on epithelial-mesenchymal transition in pancreatic cancer. Cancer Manag Res.

[21] Fan MJ, Zou YH, He PJ, Zhang S, Sun XM, Li CZ. Long non-coding RNA promotes epithelial-mesenchymal transition of cervical cancer by regulating the miR-101-3p/ZEB1 axis. Biosci Rep. 2019;39(6):BSR20181339. 10.1042/BSR20181339 PMC6549091 31092700

[22] Chen Y, Sumardika IW, Tomonobu N, Kinoshita R, Inoue Y, Iioka H, Mitsui Y, Saito K, Ruma IMW, Sato H, Yamauchi A, Murata H, Yamamoto KI3, Tomida S, Shien K, Yamamoto H, Soh J, Futami J, Kubo M, Putranto EW, Murakami T, Liu M, Hibino T, Nishibori M, Kondo E, Toyooka S, Sakaguchi M (2019). Critical role of the MCAM-ETV4 axis triggered by extracellular S100A8/A9 in breast cancer aggressiveness. Neoplasia (New York, NY).

[23] Schmittgen TD, Livak KJ (2008). Analyzing real-time PCR data by the comparative C T method. Nat Protocol.

[24] Wilusz JE, Sharp PA (2013). A circuitous route to noncoding RNA. Science..

[25] Ferlay J, Soerjomataram I, Dikshit R, Eser S, Mathers C, Rebelo M, Parkin DM, Forman D, Bray F (2015). Cancer incidence and mortality worldwide: sources, methods and major patterns in GLOBOCAN 2012. Int J Cancer.

[26] Jiang LH, Hou JC, Zhong SL, Zhou SY, Zhu LP, Li J, Wang DD, Sun DW, Ji ZL, Tang JH (2018). Circular RNA hsa_circ_0052112 promotes cell migration and invasion by acting as sponge for miR-125a-5p in breast cancer. Biomed Pharmacother.

[27] Wang S, Li Q, Wang Y, Li X, Wang R, Kang Y, Xue X, Meng R, Wei Q, Feng X (2018). Upregulation of circ-UBAP2 predicts poor prognosis and promotes triple-negative breast cancer progression through the miR-661/MTA1 pathway. Biochem Biophys Res Commun.

[28] Porter AG, Jänicke RU (1999). Emerging roles of caspase-3 in apoptosis. Cell Death Differ.

[29] Schickel R, Boyerinas B, Park S, Peter M (2008). MicroRNAs: key players in the immune system, differentiation, tumorigenesis and cell death. Oncogene..

[30] Chen K, Zhang L (2019). LINC00339 regulates ROCK1 by miR-152 to promote cell proliferation and migration in hepatocellular carcinoma. J Cell Biochem.

[31] Feng F, Liu H, Chen A, Xia Q, Zhao Y, Jin X, Huang J (2019). miR-148-3p and miR-152-3p synergistically regulate prostate cancer progression via repressing KLF4. J Cell Biochem.

[32] Ghazanchaei A, Mansoori B, Mohammadi A, Biglari A, Baradaran B (2018). Restoration of miR-152 expression suppresses cell proliferation, survival, and migration through inhibition of AKT-ERK pathway in colorectal cancer. J Cell Physiol.

[33] Zhang H, Lu Y, Wang S, Sheng X, Zhang S (2019). MicroRNA-152 acts as a tumor suppressor microRNA by inhibiting Krüppel-like factor 5 in human cervical cancer. Oncol Res.

